# Real-time transition dynamics and stability of chip-scale dispersion-managed frequency microcombs

**DOI:** 10.1038/s41377-020-0290-3

**Published:** 2020-04-03

**Authors:** Yongnan Li, Shu-Wei Huang, Bowen Li, Hao Liu, Jinghui Yang, Abhinav Kumar Vinod, Ke Wang, Mingbin Yu, Dim-Lee Kwong, Hui-Tian Wang, Kenneth Kin-Yip Wong, Chee Wei Wong

**Affiliations:** 1 Fang Lu Mesoscopic Optics and Quantum Electronics Laboratory, University of California, Los Angeles, CA 90095 USA; 2 School of Physics and The MOE Key Laboratory of Weak Light Nonlinear Photonics, Nankai University, Tianjin, China; 3 Department of Electrical, Computer and Energy Engineering, University of Colorado Boulder, Boulder, CO 80309 USA; 4 Department of Electrical and Electronic Engineering, The University of Hong Kong, Hong Kong, China; 5 Institute of Microelectronics, A*STAR, Singapore, 117865 Singapore

**Keywords:** Optics and photonics, Physics

## Abstract

Femtosecond mode-locked laser frequency combs have served as the cornerstone in precision spectroscopy, all-optical atomic clocks, and measurements of ultrafast dynamics. Recently frequency microcombs based on nonlinear microresonators have been examined, exhibiting remarkable precision approaching that of laser frequency combs, on a solid-state chip-scale platform and from a fundamentally different physical origin. Despite recent successes, to date, the real-time dynamical origins and high-power stabilities of such frequency microcombs have not been fully addressed. Here, we unravel the transitional dynamics of frequency microcombs from chaotic background routes to femtosecond mode-locking in real time, enabled by our ultrafast temporal magnifier metrology and improved stability of dispersion-managed dissipative solitons. Through our dispersion-managed oscillator, we further report a stability zone that is more than an order-of-magnitude larger than its prior static homogeneous counterparts, providing a novel platform for understanding ultrafast dissipative dynamics and offering a new path towards high-power frequency microcombs.

## Introduction

Optical frequency combs, first demonstrated in Mode-locked lasers, are unique light sources that coherently link optical frequencies with microwave electrical signals and have largely influenced studies in frequency metrology, optical clocks, precision navigation, and high-speed communication in recent decades^1^. The dissipative Kerr soliton, a local attractor that doubly balances the parametric gain and cavity loss, as well as the Kerr nonlinearity and dispersion^2^, has drawn significant attention in frequency comb studies in recent years. These investigations have led to the study of soliton and soliton molecule dynamics^3–6^. In particular, efforts in the breathing pulse evolution along the cavity length—through dispersion management—could broaden the scope of mode-locking physics and solve the high-energy pulse break-up problem induced by an excessive accumulated nonlinear phase, a serious limit in traditional cavities where pulses propagate statically^7–9^.

The miniaturization of frequency comb generation into chip-based microresonators offers an opportunity to examine dissipative Kerr soliton generation in compact footprints^10^, which not only provides a promising testbed to examine fascinating nonlinear dynamics^11^ but also offers a reliable route for frequency microcombs^12^ towards implementations in low-phase noise photonic oscillators^13^, broadband optical frequency synthesizers^14,15^, integrated dual-comb spectroscopy^16^, coherent terabit communications^17^, coherent laser ranging^18,19^, and spatio-temporal control of solitons^20,21^. In a normal procedure, dissipative soliton states can be generated when the driving laser frequency or power is tuned in the Kerr-active microresonator from blue detuning to red detuning, initiating spontaneous cavity modulation instability, followed by Turing pattern generation^22–24^, a transition into spatial-temporal chaos, and the eventual passage into the breather soliton, soliton molecules or crystals, and single soliton states. As previous studies of cavity dynamics in fiber optics could be boosted by real-time measurement technology^25–27^, the study of these abundant transition dynamics in a microcavity can benefit from a time magnifier system due to its ability to characterize non-repetitive and arbitrary waveforms in real time with sub-picosecond temporal resolution in a single shot^28,29^. Complementing the techniques of direct detection^30^ and electro-optic comb scanning^31^, temporal magnification can help surpass the electronic limits when studying microresonator dynamics at very high repetition rates of several tens of GHz or more. In steady state, static dissipative solitons have been observed and characterized in both the anomalous^32^ and normal dispersion regimes^33,34^. Dispersion-managed dissipative solitons have also been theoretically studied in Kerr active resonators^35^, including cases with shorter pulse widths for higher pulse energies. Specifically, dispersion-managed microresonators^36^ can exhibit improved output pulse stability via the reduced timing jitter converted from pulse center frequency fluctuations due to the near-zero net GVD (group velocity dispersion). In addition, a dispersion-managed dissipative Kerr soliton is more resistant to breather soliton instabilities, increasing the attainable pulse energy from a passive resonator^35^.

Here, we report the real-time transitional dynamics and enhanced stability of dispersion-managed dissipative solitons. Our oscillator is designed asymmetrically with adiabatic tapering where single-mode operation, a high quality factor, and dispersion management are attained simultaneously^36^. This design enables the enhanced stable formation of dispersion-managed dissipative solitons. Subsequently, through our ultrafast temporal magnifier (UTM) approach^29^, we are able to map—in real-time—transitional portraits of femtosecond mode-locking from noisy chaotic backgrounds. We illustrate the complex bifurcation dynamics of these tuned dissipative solitons. Third, through correlated transmission measurements, we show that our dispersion-managed dissipative solitons exist in a stability zone that is an order-of-magnitude larger than that in prior static homogenous oscillators and sustain more pulse energy.

## Results

### Dispersion-managed dissipative Kerr solitons in a tapered microresonator

**Fig. 1:**
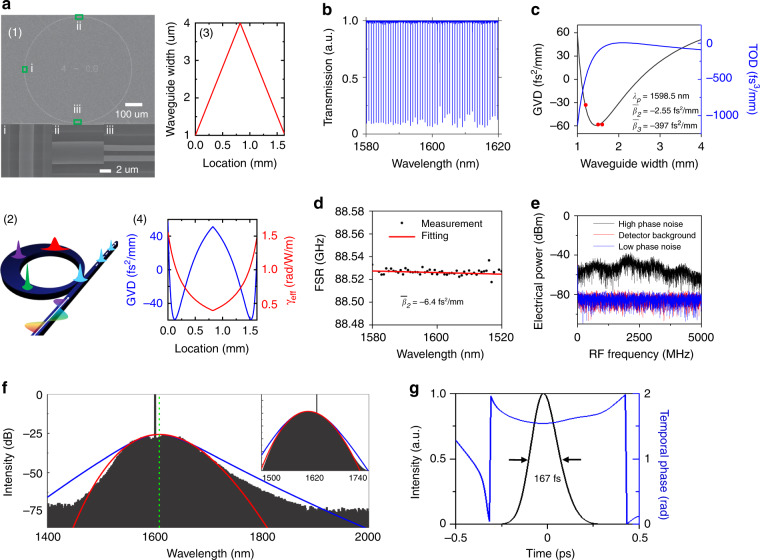
Dispersion-managed dissipative Kerr soliton generation with an adiabatically tapered Si_3_N_4_ microring. **a** (1) SEM image of the dispersion-managed microcavity. (2) Conceptual schematic of the tapered Si_3_N_4_ microring and the breathing pulse evolution along the cavity length. The varying widths of the cavity waveguide provide an oscillating group velocity dispersion (GVD) and varying nonlinear coefficient. (3) The waveguide width changes at different locations of the microcavity. (4) The GVD (blue curve) and nonlinear coefficient (red curve) at the pump wavelength (1598.5 nm) change at different locations of the microcavity. **b** Cold cavity transmission of the tapered Si_3_N_4_ microring, measured with a high-resolution coherent swept wavelength interferometer (SWI) (see Supplementary Section I). The existence of higher-order transverse modes is not observed across the wavelength region of interest. The *Q* factors and wavelength-dependent free spectral range (FSR) are determined from the transmission measurement. **c** COMSOL-modeled GVD and third-order dispersion (TOD) of the Si_3_N_4_ waveguide with respect to the waveguide width, taking into consideration both the waveguide dimensions and the material dispersion. At the pump wavelength of 1598.5 nm, the path-averaged GVD and TOD are −2.6 fs^2^ mm^−1^ and −397 fs^3^ mm^−1^, respectively. The red dots are the measured GVD for waveguides with widths of 1.2 μm, 1.5 μm, and 1.6 μm, showing good agreement with the simulation results (see Supplementary Section I). **d** Wavelength dependence of the FSR, determining the residual non-equidistance of the modes, $D = - \beta _2\omega _{FSR}^2c/2\pi n$, of 54 ± 3 kHz. The extracted GVD is anomalous at −6.4 ± 0.4 fs^2^ mm^−1^, in good agreement with the simulation result. **e** RF amplitude noise of the Kerr frequency microcomb in different states, showing the transition into the low-phase noise state with amplitude noise reaching the detector background. The 5 GHz scan range is more than 50 times the cavity linewidth. **f** The measured optical spectrum of the dispersion-managed dissipative soliton, which fits better to a Gaussian profile (red curve) than a sech^2^ profile (blue curve). The 3 dB bandwidth of the measured spectrum is 4.78 THz, and the corresponding FWHM of the transformed-limited pulse is 92 fs. Inset: simulated comb spectrum, also showing a better match with a Gaussian profile than a sech^2^ profile. **g** Pulse shape (black line) and temporal phase (blue line) retrieved from the FROG measurement. The FWHM pulse duration is measured to be 167 fs

**Fig. 2:**
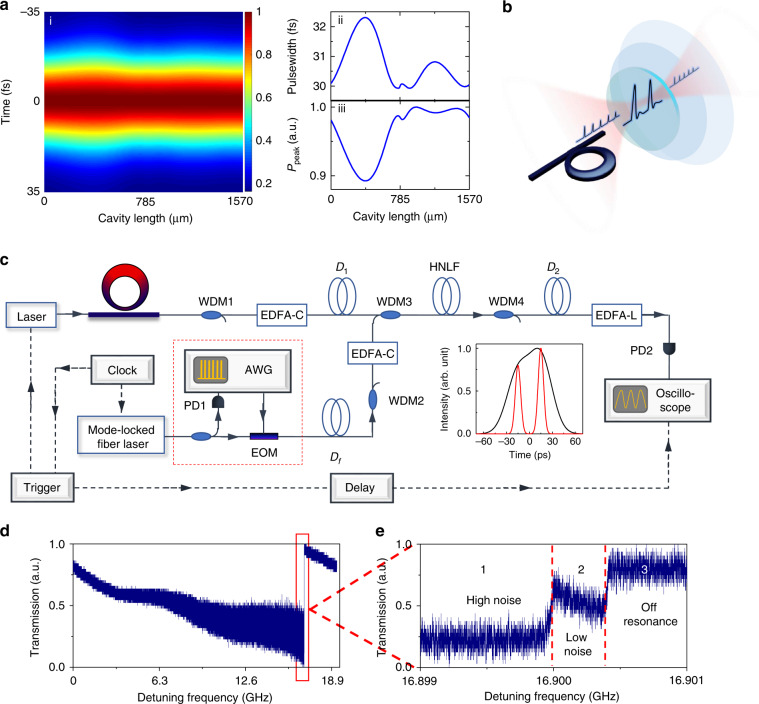
Observing the evolution and transition dynamics with an ultrafast temporal magnifier. **a**, i: NLSE-modeled dissipative Kerr soliton dynamics with an oscillating pulse width due to dispersion management. The pulse width changes along the cavity length length. ii: The variation in the FWHM of the dispersion-managed dissipative soliton along the cavity length. iii: The variation in the peak power of the dispersion-managed dissipative soliton along the cavity length. **b** Conceptual schematic of the ultrafast temporal magnifier (UTM), the time-domain counterpart of a high-speed digital microscope system. **c** Schematic setup of the UTM system. Along the fast time axis, the temporal structure of the intracavity field is magnified and captured by a real-time oscilloscope. The temporal magnification and time resolution of the UTM system are ×72 and 600 fs, respectively. Along the slow time axis, the evolution and transition dynamics are sampled optically with a stabilized femtosecond Mode-locked fiber laser. The frame rate of the UTM system is thus 250 MHz, limited by the repetition rate of the Mode-locked fiber laser. All of the electronics are commonly referenced to an Rb-disciplined crystal oscillator for accurate synchronization. Inset: two pulses separated by 30 ps, originally unresolved (black curve), are distinguishable via the UTM system. ECDL: external cavity diode laser. WDM: wavelength-division multiplexing. EDFA: erbium-doped fiber amplifier. AWG: arbitrary waveform generator. EOM: electro-optic modulator. PD: photodetector. *D*_*1*_, *D*_*2*_, and *D*_*f*_ are the dispersions for the UTMs (see Supplementary Table I). To increase the SNR and reduce the aberration, the measurement frame rate is reduced to 25 MHz by picking 1 pulse out of 10 with an EOM driven by a synchronized AWG (red dashed box). **d** Total power transmission as the pump frequency is scanned across a cavity resonance at a speed of 2.1 THz s^−1^ for an on-chip pump power of 30 dBm. The step signature is characteristic of the low-phase noise soliton state (state 2). **e** The dissipative Kerr soliton dynamics around these transmission steps are studied and portrayed with the UTM system

A scanning electron microscopy (SEM) image of the dispersion-managed microresonator is shown in Fig. 1a (1). Figure 1a (2) depicts a conceptual picture of the Si_3_N_4_ microresonator, which consists of a waveguide with varying widths to provide the oscillating group velocity dispersion (GVD) along the cavity length (a detailed design of the waveguide width is shown in Fig. 1a (3)). Figure 1a (4) shows the GVD and nonlinear coefficient varying along the cavity length. Since the effect of the varying nonlinear coefficient is negligible compared to the changing GVD, we only consider the impact of GVD management in this work. Such an oscillating GVD results in the periodic stretching and compression of dispersion-managed dissipative solitons (DM-DSs) and is more resistant to breather soliton instability, increasing the attainable pulse energy from a passive resonator^34^ (see supplementary Information Section II). In our current design, the microresonator waveguide width first changes from 1 µm at the coupling region to 4 µm in the middle of the microcavity and then changes back to 1 µm in the second half of the microcavity, resulting in a GVD oscillation from −59 fs^2^ mm^−1^ to +58 fs^2^ mm^−1^ (see supplementary Information Section I for details). We solve a system consisting of the cavity coupling equation and the nonlinear Schrödinger equation numerically to describe the DM-DS formation physics:

$$\left\{ {\begin{array}{*{20}{c}} {A^{m + 1} = \sqrt {TP_{in}} + \sqrt {1 - T} A^m{\mathrm{exp}}\left( { - j\delta } \right)} \\ {\frac{\partial }{{\partial z}}A^m = - \frac{\alpha }{2}A^m - \frac{j}{2}\beta _2(z)\frac{{\partial ^2}}{{\partial t^2}}A^m + j\gamma (z)\left| {A^m} \right|^2A^m} \end{array}} \right.$$

where *m*, *β*_*2*_, *α*, *T*, *γ*, and *δ* are the number of roundtrips, GVD, cavity loss, coupling loss, Kerr nonlinear coefficient, and pump-resonance detuning, respectively. The cavity length is discretized into a total of 120 steps, and at each step, *β*_*2*_ and *γ* are re-evaluated based on the local waveguide geometry. Figure 2a plots the numerically solved DM-DS evolution within the microcavity. The asymmetry of the pulse width and peak power shown in Fig. 2a ii and iii is due to the chirp change (see the detailed simulation result and discussion in Supplementary Section II). For each round trip, the DM-DS experiences a cycle of stretching and compression between 32.3 fs and 29.9 fs (Supplementary Fig. 6).

The Si_3_N_4_ microresonator design not only provides an oscillating GVD along the cavity length but also suppresses higher-order mode families, which suppresses the mode-crossing-induced perturbation to dissipative soliton generation, leaving only the rare mode-crossing caused by TE-TM mode hybridization from the fabrication imperfection^35^. Both the bus waveguide and the cavity waveguide in the coupling region are designed to be strictly single-mode, thereby ensuring selective excitation of the fundamental mode and suppressing other transverse mode families, leading to near-ideal coupling^37^. The cavity transmission around the pump mode is plotted in Fig. 1b, with no observable higher-order transverse modes in the transmission spectrum. Near-critical coupling is attained, with a loaded *Q* of 1.9 × 10^6^ and a cavity loading of 90% for the pump mode (see Supplementary Information Section I for details). The microresonator free spectral range is measured to be ≈88 GHz. The GVD and TOD shown in Fig. 1c are calculated with a commercial full-vectorial finite-element-method solver (COMSOL Multiphysics), taking into consideration both the cavity geometry and the material dispersion. The path-averaged GVD is slightly anomalous at −2.6 fs^2^ mm^−1^, leading to intracavity pulse dynamics in the stretched-pulse regime. Operation in the stretched-pulse regime with near-zero GVD has been demonstrated to be beneficial in femtosecond Mode-locked fiber lasers for achieving a narrow linewidth, low phase noise, and attosecond timing jitter^38^, which are important merits for advancing microwave photonics^39^ and coherent pulse synthesis^40^. A similar timing jitter reduction from decreasing the net cavity dispersion is also theoretically predicted in Kerr-active resonators^41^. To verify the near-zero path-averaged GVD, high-resolution frequency-comb-assisted diode laser spectroscopy^42^ is employed to characterize the cold cavity properties of our designed Si_3_N_4_ microresonator. With active control of the on-chip temperature, passive shielding against acoustic noise and an absolute wavelength calibration with the hydrogen cyanide gas standard, the method provides a GVD accuracy of 0.4 fs^2^ mm^−1^, determined as the standard deviation calculated from 10 independent measurements (see Supplementary Information Section I for details). The mean value of the net cavity GVD from the 10 measurements is −6.4 fs^2^ mm^−1^ (Fig. 1d).

Tuning the pump frequency into the cavity resonance from the blue side with a scan speed of 3.5 THz s^−1^, a stable DM-DS can be observed, with a typical optical spectrum shown in Fig. 1f. The 3 dB bandwidth of the measured spectrum is 4.78 THz, and the transform-limited FWHM pulse duration is 92 fs. Notably, the measured DM-DS spectrum fits well with a Gaussian profile (red line) rather than the squared hyperbolic shape (blue line), indicative of stretched-pulse operation^35^. The inset plots the numerically simulated comb spectrum, which also shows a better match with a Gaussian profile than a sech^2^ profile. Furthermore, the TOD effect is augmented due to the near-zero GVD, resulting in the observed asymmetry in the optical spectrum and carrier frequency shift (green dashed line) from the pump. A stable pulse train and low-noise operation is confirmed by an RF amplitude noise spectrum measurement (Fig. 1e), showing that the noise level approaches the detector background, and a frequency-resolved-optical-gating (FROG) measurement (Fig. 1g), showing consistently low retrieval errors below 10^−2^. A singlet DM-DS pulse with a negative chirp, in qualitative agreement with the numerical simulation, is retrieved from the FROG spectrogram. We note that a quantitative comparison cannot be made due to the limited bandwidth of the C-band Er-doped fiber amplifier used in our FROG measurement (see Methods and Supplementary Information Section III for details).

### Real-time dynamics measurement with an ultrafast temporal magnifier

**Fig. 3:**
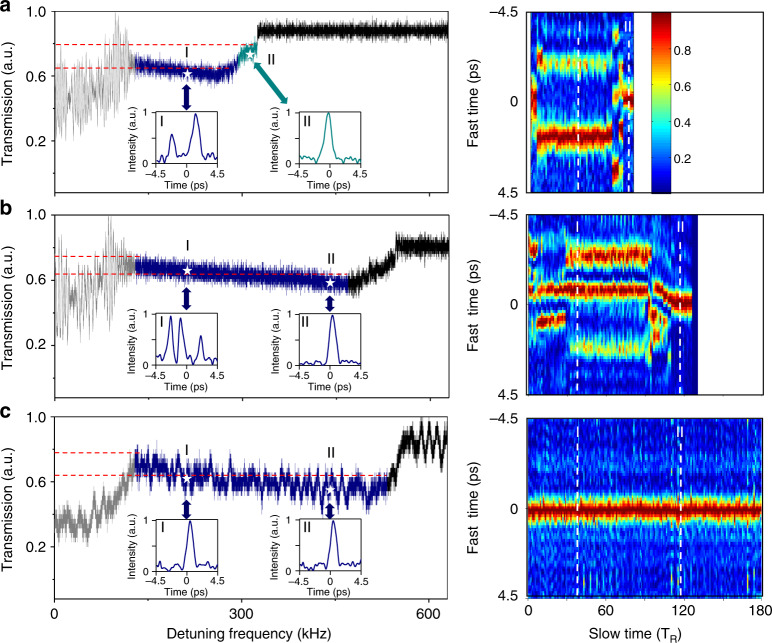
UTM-enabled comparison of the stability zone and temporal dynamics between static and dispersion-managed dissipative Kerr solitons. **a** Total power transmission (left panel) and the 2D evolution portrait (right panel) of static soliton formation in a homogenous microresonator with a measured GVD of −33.1 fs^2^ mm^−1^. A single soliton is only observed in the last transmission step and remains stable for the pump resonance detuning range of 30 kHz, where the cavity loading is reduced to 30%. The on-chip pump power is 30 dBm. **b**, **c** Total power transmission (left panel) and the 2D evolution portrait (right panel) of dispersion-managed soliton formation in the tapered microresonator, showing increased stability zones at a higher pump power than static solitons. In panel **b** with an on-chip pump power of 30 dBm, transitions from a chaotic oscillation to a multiple soliton state and eventually to a single soliton state are also observed. However, unlike the static soliton formation shown in **a** the transition from multiple to single soliton states does not require an ejection of excessive intracavity power. Instead, a monotonic increase in the cavity loading from 38% to 52% is measured along the gradual transition into a single soliton state. In panel **c** as the on-chip pump power increases to 32 dBm, a single soliton state can persist across the whole transmission step and remain stable for a pump resonance detuning of 400 kHz, while the cavity loading monotonically increases to 57%. At this pump power level, a low-noise stable soliton state is not observed in a homogeneous microresonator. Insets: measured pulse shapes at the pump resonance detuning denoted by the white dashed lines in the 2D mappings

While the temporal structure of the intracavity field is detailed at the sub-picosecond time scale, the evolution and transition dynamics are portrayed at a much slower sub-microsecond time scale, which is associated with the cavity photon time of the microresonator. The orders-of-magnitude difference in the time scale between the two time dimensions poses an experimental challenge for capturing a comprehensive picture of the dynamics. Here, we demonstrate that UTMs are invaluable solutions that can fully characterize the evolution and transitional dynamics of dissipative Kerr solitons. A UTM is the time-domain counterpart of a high-speed digital microscope system, utilizing the space-time duality principle where diffraction in space and dispersion in time share the same mathematical expression^29^ (Fig. 2b). Incorporating suitable GDDs (*D*_*1*_ and *D*_*2*_) before and after the four-wave mixing stage in a highly nonlinear fiber (HNLF), a temporal magnification of ×72 and a time resolution of 600 fs are attained in our UTM, enabling a detailed depiction of the dissipative soliton’s temporal structure. The evolution and transition dynamics, on the other hand, are sampled optically with a synchronized and stabilized femtosecond Mode-locked fiber laser (MenloSystems GmbH). The frame rate of the first UTM is 250 MHz, higher than the cavity resonance linewidth of 100 MHz, and is determined by the laser repetition rate stabilized to an Rb-disciplined crystal oscillator (Fig. 3c, see Methods and Supplementary Section IV). At a frame rate of 25 MHz for the second UTM and with a 190 ps single-shot record length, we can record a complete intracavity field of 16 roundtrips in real time, but we miss the information between each measurement. Then, multiple frames are stitched to reconstruct the soliton evolution through the whole transmission step. As long as the intracavity field pattern evolves much slower than 25 MHz, our technique could provide valuable information about the intracavity waveform evolution.

Figure 2d shows the total transmission of the microresonator with respect to the pump resonance detuning when the pump frequency is scanned across the resonance at a speed of 2.1 THz s^−1^. The transmission deviates from a Lorentzian lineshape and follows a triangular profile defined by the combined effect of the thermal and nonlinear resonance shifts, resulting in optical bistability that eventually leads to dissipative Kerr soliton formation. At the edge of the resonance, multiple discrete transmission steps are observed, which have been identified as important attributes of dissipative Kerr soliton (Fig. 2e). As solitons are formed, excessive optical power is ejected from the cavity, resulting in a stepwise increase in the total transmission^10^. To compare the difference in the transition dynamics and stability zone between static and dispersion-managed dissipative Kerr solitons, we synchronize both the transmission and UTM measurements with pump frequency scanning and focus on the dynamics around these transmission steps. A representative result of static soliton formation in a homogenous microresonator is summarized in Fig. 3a. The Si_3_N_4_ microresonator consists of a 1.2 × 0.8 μm^2^ uniform waveguide, with a loaded *Q* of 1.5 × 10^6^, a cavity loading of 86%, and a measured GVD of −33.1 fs^2^ mm^−1^ (Fig. 1c). At the first transmission step where the cavity loading decreases to 45% (corresponding to a transmission of 55%), a doublet soliton state is reached and remains stable for a 160 kHz change in the pump resonance detuning before the second transmission jump. A further reduction in the cavity loading to 30% results in the formation of a singlet soliton state, which nevertheless only exists in a small stability region of 30 kHz. An on-chip pump power of 30 dBm is used in this example, but similar behavior is noted for pump powers of 24 dBm and 27 dBm. However, no stable soliton states are observed from the UTM when the on-chip pump power exceeds 30 dBm.

## Discussion

In comparison, representative results of dispersion-managed soliton formation with distinctly different features are summarized in Fig. 3b, c. First, a stable singlet soliton is observed at both power levels of 30 dBm (Fig. 3b) and 32 dBm (Fig. 3c), with a better success rate in achieving the singlet soliton state at higher power. The on-chip pump power of 32 dBm is limited by the available power of our high-power erbium-doped fiber amplifier. Second, while the transition from chaos to low-noise stable solitons is still associated with a transmission step, the transition from a multiple soliton state to a singlet soliton state no longer results in a further ejection of excessive intracavity power. Rather, monotonic increases in the cavity loading from 38% to 52% (Fig. 3b) and from 36% to 57% (Fig. 3c) are observed. When the on-chip pump power is 30 dBm, triplet solitons are first observed after the transmission step (Fig. 3b–I), and their interaction gradually results in a transition to a singlet soliton state at the end of the transmission step (Fig. 3b–II). Both characteristics illustrate the advantage of the dispersion-managed Kerr soliton in concentrating more energy into a singlet soliton pulse. Furthermore, the stability zone of dispersion-managed Kerr soliton states including both the multiple and singlet soliton cases are extended to 340 kHz (Fig. 3b) and 400 kHz (Fig. 3c), more than double the stability zone of a static soliton. In particular, at a higher on-chip pump power of 32 dBm, the singlet soliton state can persist across the whole transmission step (Fig. 3c), showing a remarkable increase in the singlet soliton stability zone by more than an order of magnitude (400 kHz as opposed to 30 kHz) at a higher pump power (see summary in Supplementary Section V).

**Fig. 4:**
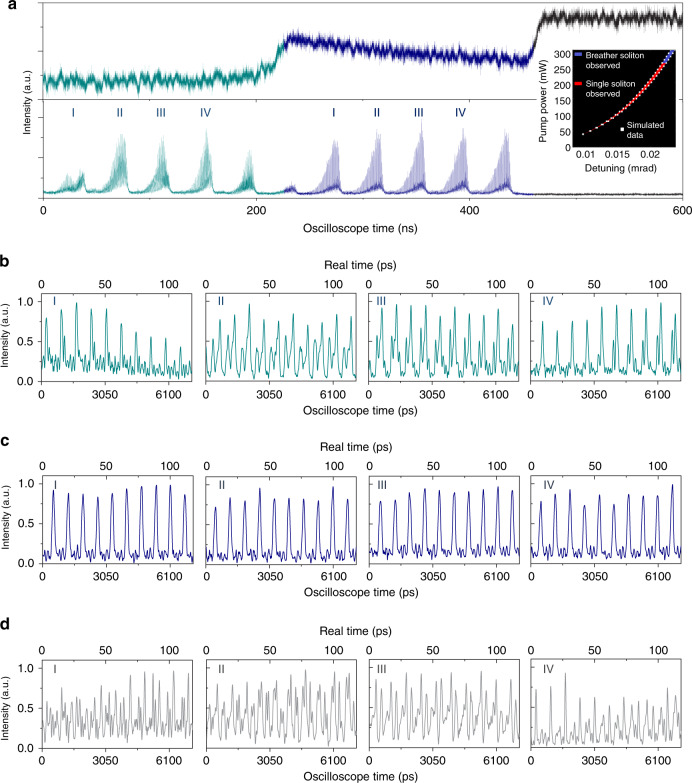
Transition dynamics of a dispersion-managed dissipative Kerr soliton. Using a modified UTM with an improved SNR and reduced aberration, the evolution of not only the low-noise stable solitons but also the chaotic oscillation before the transition can be characterized. The on-chip pump power is 32 dBm, and the scan speed is 2.8 THz s^−1^. **a** Total power transmission (top panel) and magnified optical waveforms on the real-time oscilloscope (bottom panel). For the case where the singlet soliton state persists across the whole transmission step (**b**), the temporal pulse structure is still discernible before the transition despite intense and rapid soliton interaction dynamics (**c**). We note that real time is the oscilloscope time divided by M_UTM_. Inset of **a**: stability modeling of the dispersion-managed microresonator. **d** With the SNR-improved UTM, chaotic states could also be successfully recorded

The generation of this particularly stable singlet soliton state is found to be correlated with a cleaner transmission curve before the transition (gray region in Fig. 3), with the root mean squared fluctuation decreasing from 20% to 10%. To probe the real-time dynamics in this potentially chaotic regime, the UTM is slightly modified to increase the signal-to-noise ratio and reduce the TOD-induced aberration at the cost of lowering the measurement frame rate (red dashed box in Fig. 2c). While the overall system GVD is increased by more than a factor of 5, the TOD is minimized by a proper combination of a dispersion-compensating fiber and a non-zero-dispersion-shifted fiber (see Supplementary Section IV). The output from the stabilized femtosecond Mode-locked fiber laser comb, for optical sampling along the slow time axis, is pulse-picked with an electro-optic modulator (EOM) to reduce the repetition rate down to 25 MHz. An arbitrary waveform generator (AWG), electronically synchronized to the Mode-locked pulse train, serves effectively as a high-quality frequency divider and drives the EOM for pulse picking with a dynamic extinction ratio of more than 20 dB. With the modified UTM, we are able to measure the evolution of not only the low-noise stable solitons but also the chaotic oscillations before the stable transition (Fig. 4). In the case where the singlet soliton remains stable across the whole transmission step (Fig. 3c), the temporal structure before the transition appears to be less chaotic, resulting in a cleaner transmission curve. Despite intense and rapid soliton interaction dynamics (Fig. 4b), a clear temporal pulse structure is still observed, which is quite different from the chaotic state (Fig. 4d). The inset in Fig. 4a shows a stability analysis of the dispersion-managed oscillator, showing that such a design could enlarge the single soliton zone with a high pump power (see Supplementary Information Section II for detailed discussion).

In summary, we report observations of dispersion-managed dissipative solitons in an adiabatically tapered Si_3_N_4_ oscillator. The tuned GVD along the cavity length manifests itself in a Gaussian-like optical spectrum that has a more equalized pump-matching comb power than a sech^2^ spectral shape, which is advantageous for applications such as astrospectrograph calibration and high-capacity coherent communication. Stretched-pulse operation with near-zero net cavity GVD is beneficial for reducing the timing jitter of frequency microcombs and advancing on-chip microwave photonics and precision metrology. We apply UTM metrology to observe and study the evolution dynamics of our dispersion-managed dissipative solitons. With this new approach, we portray the soliton transition dynamics and show that these dispersion-managed dissipative solitons exist in a stability zone that is an order-of-magnitude larger at a higher pump power than their static counterpart in traditional homogeneous oscillators and sustain more pulse energy. The dispersion management demonstrated by the design of the waveguide geometry can be generalized and extended to other material systems, such silicon^43^, high-refractive-index glass^44^, aluminum nitride^45^, AlGaAs^46^ and lithium niobite^47^, and unconventional spectral ranges, such as the ultraviolet^48^ and mid-infrared ranges^49^; the same principle can also be applied to other types of microresonators by engineering whispering gallery mode cavity structures^50^. The dispersion-managed dissipative soliton microresonator provides a novel platform for understanding ultrafast dissipative cavity dynamics and offers a new path towards high-power frequency microcombs.

## Methods

### Device fabrication

The microresonator was fabricated with CMOS-compatible processes: first, a 5-μm-thick under-cladding oxide was deposited via plasma-enhanced chemical vapor deposition to suppress the substrate loss. An 800-nm-thick Si_3_N_4_ layer was then deposited via low-pressure chemical vapor deposition, patterned by optimized deep-ultraviolet lithography, and etched via optimized reactive ion dry etching. A sidewall angle of 88° was determined using transmission electron microscopy. Annealing at a temperature of 1150 °C was then applied to the chip for 3 h to reduce the waveguide propagation loss. Finally, the dispersion-managed microresonator was over-cladded with a 3-μm-thick oxide layer, deposited initially with LPCVD (500 nm) and then with PECVD (2500 nm). Adiabatic mode converters were implemented on the side of the chip to improve the coupling efficiency from the free space to the bus waveguide with less than 3 dB of coupling loss per facet.

### FROG measurement of the dispersion-managed dissipative soliton

We measured the pulse duration via sub-femto-joule sensitive second-harmonic-generation (SHG) non-collinear frequency-resolved optical gating (FROG). Since the comb-line power of the soliton state was too low, a C-band erbium-doped fiber amplifier (EDFA-C) was applied after the comb was filtered out by a low-pass filter (LPF). The dispersion introduced by the amplifier and SMF connected the soliton generation setup, and the second-harmonic generation (SHG) setup was carefully compensated by dispersion compensation fiber (DCF). Then, the SHG was sent into a Horiba 1000M series II spectrometer for the FROG measurement. Then, commercial FROG retrieval software (Swamp Optics, LLC.) was utilized to retrieve the temporal profile and phase information of the soliton state. The FROG error was typically kept below 0.01. To achieve the FROG measurements, the specific soliton state was maintained for at least 30 min to an hour.

### Measurement of the real-time dynamics

We used the ultrafast temporal magnifier (UTM) to measure the 2D mapping of the dissipative Kerr soliton dynamics. Our UTM was implemented though four-wave mixing (FWM) in a 50-m highly nonlinear fiber (HNLF). The HNLF (OFS) had a zero-dispersion wavelength (ZDW) of 1556 nm and a dispersion slope of 0.019 ps/(nm^2^·km). The nonlinear coefficient was 11.5 W^−1^ km^−1^. The FWM pump was derived from a stabilized femtosecond Mode-locked fiber laser, and its spectral component from 1554 nm to 1563 nm was first filtered out by wavelength-division multiplexing (WDM2) and subsequently amplified by a C-band erbium-doped fiber amplifier (EDFA-C) to 50 mW. Before the signal and the pump were combined via WDM3 into the FWM stage, they were chirped by *D*_*1*_ and *D*_*f*_, respectively, through two spools of SMF-28. The generated idler was filtered out via WDM4 and then chirped by the output dispersion *D*_*2*_ through a spool of DCF. Finally, the filtered and chirped idler was amplified by an L-band erbium-doped fiber amplifier (EDFA-L) to 100 μW before it was sent to a 25 GHz amplified photodetector (PD2) and an 80 GS s^−1^ real-time oscilloscope for detection. For the correlation measurements, the ECDL laser frequency scan and the oscilloscope data acquisition system were synchronized using a multi-channel pattern generator. All of the electronics were commonly referenced to an Rb-disciplined crystal oscillator for accurate timing.

## Supplementary information

Supplementary Information
