# Supplementary material for: Real-time transition dynamics and stability of chip-scale dispersion-managed frequency microcombs
Source: Light Sci Appl. 2020 Apr 3;9:52. doi: 10.1038/s41377-020-0290-3 (PMC7118405; doi:10.1038/s41377-020-0290-3)
Supplement: Supplementary file 1 — Supplementary Information [file 41377_2020_290_MOESM1_ESM.docx]

**Supplementary Information**

**Real-time transition dynamics and stability of chip-scale dispersion-managed frequency microcombs**

Yongnan Li^1,2,†^, Shu-Wei Huang^1,3,†,^**^＊^**, Bowen Li^4,†^, Hao Liu^1,†,*^, Jinghui Yang^1^, Abhinav Kumar Vinod^1^, Ke Wang^2^, Mingbin Yu^5^, Dim-Lee Kwong^5^, Hui-Tian Wang^2^, Kenneth Kin-Yip Wong^4^, and Chee Wei Wong^1,^**^＊^**

^1^ Fang Lu Mesoscopic Optics and Quantum Electronics Laboratory, University of California, Los Angeles, CA 90095, USA.

^2^ School of Physics and The MOE Key Laboratory of Weak Light Nonlinear Photonics, Nankai University, Tianjin, China.

^3^ Department of Electrical, Computer & Energy Engineering, University of Colorado Boulder, Boulder, Colorado 80309, USA.

^4^ Department of Electrical and Electronic Engineering, The University of Hong Kong, Hong Kong, China.

^5^ Institute of Microelectronics, A*STAR, Singapore 117865, Singapore.

^†^ these authors contributed equally to this work

**^＊^** corresponding author e-mail: shuwei.huang@colorado.edu; haoliu1991@ucla.edu; cheewei.wong@ucla.edu

This Supplementary Information consists of the following sections:

1. **Characterization of the stretched-pulse Si_3_N_4_ microcavity**
2. **Simulation of the dispersion-managed dissipative Kerr soliton**
3. **Pulse characterization with frequency-resolved optical gating**
4. **Ultrafast temporal magnifier**
5. **Stability zone comparison between DM-DS and DS generated in conventional uniform microcavity**
6. **Effect of periodically-varying nonlinearity**

**Supplementary Section I. Characterization of the stretched-pulse Si_3_N_4_ microcavity**

#

**Figure S1 | Properties of the tapered microring. a,** Transverse distributions of the TE_00_ mode fields along the tapered microring. Red, yellow, blue and green dotted line represents the waveguide width a 1 μm, 2 μm, 3 μm, and 4 μm respectively. **b,** The microscopy picture of the tapered microring. **c,** The variation of the waveguide width along the cavity. The circumference of the tapered microring is 1570 μm. **d,** Cold cavity transmission of the tapered Si_3_N_4_ microring, measured with high resolution coherent swept wavelength interferometer (SWI) (Figure S4). Existence of higher order transverse modes is not observed across the wavelength region of interest. *Q* factors and wavelength-dependent free spectral range (FSR) are determined from the transmission measurement. **e,** The resonance at pump wavelength (1598.5 nm).

Figure S1a shows the COMSOL modeled field distributions of the fundamental TE_00_ modes along the adiabatically tapered microring. Modeling is performed on 50 nm triangular spatial grid with perfectly-matched layer absorbing boundaries and 5 pm spectral resolution. The microring has a diameter of 500 μm and tapered widths from 1 μm to 4 μm (Figures S1b and S1c) to provide variation in effective mode index, *n*_eff_, and dispersion management along the cavity. Figures S1d and S1e show the measured transmission of the microring. Close to critical coupling is attained, with a loaded *Q* of 1.9×10^6^ and a cavity loading of 90% at the pump mode (1598.5 nm).

Since the microring diameter is sufficiently large, the bending loss and the bending dispersion of the resonator waveguide are negligible in our microring resonators. Using the 4^th^ order accurate central finite difference method, we numerically evaluate GVD and TOD in accordance with formulas$GVD=\frac{\lambda^{3}}{2\pi c_{0}^{2}}\frac{d^{2}}{d\lambda^{2}}n_{eff}$and$TOD=-\frac{\lambda^{4}}{4\pi^{2}c_{0}^{3}}\left( \lambda\frac{d^{3}}{d\lambda^{3}}n_{eff}+3\frac{d^{2}}{d\lambda^{2}}n_{eff} \right)$ (Figures S2a and S2d). For the pump wavelength (1598.5 nm), the GVD of the fundamental mode (TE_00_) of the microcavity proceeds from normal to anomalous, then back to normal when the waveguide width adiabatically increases from 1 μm to 4 μm, as shown in Figure S2b. When light propagates through the tapered microring, the path-average dispersion, *D*_ave_, is an arithmetic mean of dispersions in each segment:

$D_{ave}=\frac{1}{C}\int_{resonator} D\left(\lambda,s\right)ds$ (S1)

where *C* is the total length (*i.e.* circumference) of the microcavity; *D (λ, s)* is the dispersion at wavelength *λ*, and segment at position *s.* The increment *ds* is set as 400 nm. Continuous function of the GVD and TOD with respect to waveguide width and length of the microcavity are obtained using a cubic spline interpolation of the COMSOL modeled discrete data points (blue and red line in Figures S2b and S2e). The fitted functions are then used to calculate the path-averaged dispersions based on equation (S1). The path-averaged GVD and TOD of the tapered microring at pump wavelength are -2.6 fs^2^ mm^-1^ and -397 fs^3^ mm^-1^ respectively.

**Figure S2 | Simulated GVD and TOD of the tapered microring for the fundamental mode TE_00_. a and d,** The dispersions for different waveguide widths. **b and e,** The dispersions at pump wavelength (1598.5 nm) changed with the waveguide width of the microcavity. **c and f**, The dispersion at pump wavelength (1598.5 nm) changed with the length of the microcavity.

Figure S3 shows the high-resolution swept wavelength interferometer, which is applied for initial dispersion measurement. The microcavity transmission, from which *Q* factor and FSR values are determined (Figures S1d and S1e), is measured using a tunable laser swept through its full wavelength tuning range at a tuning rate of 60 nm s^-1^. For absolute wavelength calibration, 1% of the laser output was directed into a fiber coupled hydrogen cyanide gas cell (HCN-13-100, Wavelength References) and then into a photodetector (PD_REF_). The microcavity and gas cell transmissions are recorded simultaneously during the laser sweep by a data acquisition system whose sample clock is derived from a high speed photodetector (PD_MZI_) monitoring the laser transmission through an unbalanced fiber Mach-Zehnder Interferometer (MZI). The MZI has a path length difference of approximately 40 m, making the measurement optical frequency sampling resolution 5 MHz.

**Figure S3 | Swept wavelength interferometer for dispersion measurement.** The swept input laser is clocked with a highly-imbalanced MZI with 5 MHz optical frequency sampling resolution, and referenced against the optical transitions of an HCN reference gas cell. FC: fiber coupler; FPC: fiber polarization controller; PBS: polarization beam splitter; PD: photodetector; HCN: hydrogen cyanide.

The absolute wavelength of each sweep is determined by fitting 51 absorption features present in the gas cell transmission to determine their subsample position, assigning them known traceable wavelengths and calculating a linear fit in order to determine the full sweep wavelength information. Each resonance is fitted with a Lorentzian line shape and the dispersion of the microring resonator is then determined by analyzing the wavelength dependence of the FSR.

**Figure S4 | Measured dispersions of the homogeneous microcavities. a**, For the microring made of uniform 1.2 μm wide waveguide, the measured non-equidistance of the modes is 250 ± 6 kHz. **b**, For the microring made of uniform 1.5 μm wide waveguide, the measured non-equidistance of the modes is 762 ± 6 kHz. **c**, For the microring made of uniform 1.6 μm wide waveguide, the measured non-equidistance of the modes is 765 ± 7 kHz. All measurements are in good agreements with the simulated dispersions.

Figure S4 shows the measured wavelength dependence of the FSR for three homogenous microcavities with uniform waveguide widths of 1.2 μm, 1.5 μm, and 1.6 μm. The mean GVD values extracted from non-equidistance of the modes and calculated from 10 independent measurements, are -33.1 fs^2^ mm^-1^, -58.3 fs^2^ mm^-1^, and -58.3 fs^2^ mm^-1^ respectively. Measurements of the dispersion are in good agreement with the simulations (red dots in Fig. 2a).

**Figure S5 | Menlo-comb assisted dispersion measurement.** BP1: 70 MHz, BP2: 35 MHz. FC: fiber coupler; FPC: fiber polarization controller; PBS: polarization beam splitter; PD: photodetector; BP: band-pass filter.

In order to further confirm the close-to zero net cavity dispersion of our design, we utilize a second dispersion measurement method, frequency comb assisted diode laser spectroscopy, shown in Figure S5. A broadband tunable laser with tuning range of 80 nm around 1580 nm is used to generate RF beat notes with an erbium-doped-fiber-laser-based frequency comb (MenloSystems GmbH), which has repetition rate *f*_rep_ = 250 MHz, centered at 1560 nm, with accuracy of 10^-14^ and stability of 5×10^-13^ at 1 second. And its *f*_ceo_ is fully locked to an optical reference, which means all the comb modes are fixed at well-defined frequencies. The beat notes are detected by a photodetector (PD_REF_) with bandwidth of 150 MHz. Then the beat signal is selected by two narrow band-pass filters, centered at 35 MHz and 70 MHz, respectively (bandwidth of 2.3 MHz), which gives out 4 frequency markers in each frequency comb line interval. By setting the scanning speed of the diode laser at 40 nm s^-1^, the total 155,000 frequency markers are recorded by two ports of a high speed four-channel oscilloscope supporting 31,250,000 data points per channel, leading to a resolution of 352 kHz per data point. The remaining two channels are used for recording the transmission of the microcavity and 51 absorption lines of HCN gas cell. Then all the markers are located through an adaptive peak-finding algorithm, and a spline interpolation of the markers is used to retrieve the instantaneous frequency of the diode laser, while one absorption line of the gascell is used to calibrate the absolute frequency. Then the dispersion of the microring resonator is again determined by analyzing the wavelength dependence of the FSR. The mean value of the net cavity GVD of the tapered microring from the 10 measurements is −6.4 fs^2^ mm^-1^ (Fig. 2b).

**Supplementary Section II. Simulation of the dispersion-managed dissipative Kerr soliton**

Figure S6 summarizes the simulation results showing the intracavity evolution of pulse duration Δτ and the stable femtosecond pulse train coupled out of such stretched-pulse microcavity. We use the varied GVD (Figure S2c) and TOD (Figure S2f) to simulate the pulse dynamics. The circumference of the tapered-microcavity is finely divided into 120 segments with different GVD and TOD value, modelled in COMSOL, for different waveguide width, and the GVD and TOD is assumed uniform for each step. 2,001 modes centered at the pump are incorporated [S1] in the NLSE modeling. We also note that the mean-field approach could be examined for this cavity, even with the dispersion modulation [S2, S3, S4], which would simplify the computational approach.

Our nonlinear simulation starts from vacuum noise and is run for 1.5 × 10^5^ roundtrips until the solution reaches the steady state. For every round-trip, the dispersion-managed dissipative soliton (DM-DS) experiences a full cycle of stretching and compression between 29.9 fs and 32.3 fs (Figure S6a). Evanescent coupling occurs at position 0, and a stable 30.05-fs pulse train is coupled out of the stretched-pulse microcavity, and Figure S6c shows the 10 consecutive pulses of the stable 30.5-fs pulse train coupled out of the microcavity. The output pulse train is stable even though the intracavity pulse is breathing.

**Figure S6 | The simulations of the dispersion-managed dissipative soliton on silicon nitride tapered microring. d,** Temporal intensity and phase at different positions in the cavity. The maximum Δτ of the pulse is 32.3 fs (at one fourth of the length of the cavity) and the minimum Δτ of the pulse is 29.93 fs (at around half of the length of the cavity). **b**, The stable output of the 30.05 fs pulse train. c, NLSE modeled dissipative Kerr soliton dynamics with oscillating pulse duration due to dispersion management in a 19 GHz microcavity. **d,** The variation of the pulse duration Δτ of the dispersion-managed dissipative soliton along the cavity.

The dynamic range of pulse stretch in the 88 GHz dispersion-managed tapered microcavity is 1.08. The reason for such small pulsewidth variation (2.4 fs) is two-fold. One reason is that the pulse experiences negative chirp, zero chirp and positive chirp in a single roundtrip, which also causes the asymmetry in Figure 2a i&ii (the detailed temporal phase variation of the pulse is shown in the gif file of supplementary material). The other reason is that the cavity length is relatively small, so the accumulated chirp is not enough for larger pulsewidth variation. To explain this, we apply the same cavity design in a larger cavity (8.1 mm, 19 GHz FSR). The simulated results are shown in Figure S6c & 6d. The dynamic range of dynamic range of pulse stretch in the 19 GHz dispersion-managed taper microcavity is 1.17 and the pulsewidth variation range is 11 fs.

In order to theoretically study the enlarged soliton stability zone of dispersion-managed tapered microcavity, Numerical simulations of both dispersion-managed microcavity and uniform microcavity are performed to map out the stability zone of the solitons. The maps the soliton characteristic step zone with pump power vs. detuning, of both cavities is illustrated in Figure S7. 10,000 roundtrips with a detuning step size of 10 µrad are performed for each pump power parameter. The white squares are simulation result we obtained. Then spline interpolation is applied to map the stability region. The black region shows chaotic state or off-resonance, the red region shows in such detuning step size, single soliton is successfully observed, and the purple region shows that no single soliton is observed but only single breather soliton is observed. In dispersion-managed resonator, single soliton is still successfully observed when the pump power is up to 260 mW while single soliton can only be observed below 80 mW in uniform resonator. In addition, the highest attainable pulse energy from single soliton in dispersion-managed microresonator is calculated 50% hihger than the uniform microresonator case. Although uniform resonator has larger stability region, the tapered resonator is more resistant to breather soliton instability in higher pump power. It means that dispersion-managed tapered resonator could effectively enlarge single soliton stability zone in higher pump power, which matches our experimental observation.

**Figure S7 | Numerical simulation of soliton stability zone in dispersion-managed tapered cavity and uniform cavity.** **a,** Soliton characteristic step mapping of dispersion-managed tapered cavity. **b,** Temporal waveform of single soliton and the intracavity power along 10,000 roundtrip times in tapered cavity. **c,** Temporal waveform of single breather soliton and the intracavity power along 10,000 roundtrip times in tapered cavity. The breathing frequency is around 126 MHz. **d,** Soliton characteristic step mapping of uniform cavity with GVD of [Hao: insert here?]. **e,** Temporal waveform of single soliton and the intracavity power along 10,000 roundtrip times in uniform cavity. **f,** Temporal waveform of single breather soliton and the intracavity power along 10,000 roundtrip times in uniform cavity. The breathing frequency is around 126 MHz. **i** is temporal waveform and **ii** is intracavity power along roundtrips. The black region is no-soliton zone. The red region shows single soliton observed in a 10 µrad detuning step. The purple region shows no single soliton observed in a 10 µrad detuning step, where only single breather soliton is observed.

**Supplementary Section III. Pulse characterization with frequency-resolved optical gating**

Figure S8 shows the data of FROG measurement. The retrieved mapping looks highly alike the original measurement after de-noising, showing a FROG retrieval error at 0.01. Since the 3-dB bandwidth of the filtered and amplified comb is ≈ 30 nm centered at 1560 nm, the retrieved ≈ 167 fs FWHM pulse duration shows a close-to-transform-limited Gaussian pulse. The residual chirp *q* is determined to be -0.1672, showing a slightly negative chirp, while, from simulation, the chirp q at the coupling region is -0.1325. Such small difference is inevitable due to limited combination of single mode fiber and DCF of the fiber link.

**c**

Intensity (a.u.)

Temporal phse (rad)

**167 fs**

Time (ps)


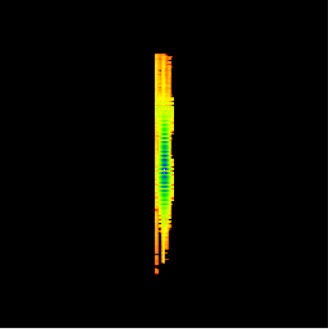

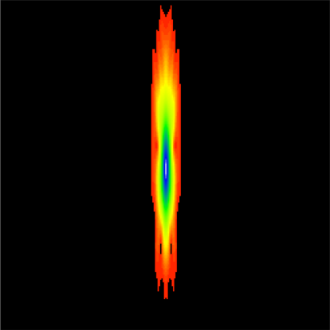


768

778

788

0

10

20

0

10

20

Delay (ps)

Delay (ps)

**a**

**b**

**Figure S8 | Frequency-resolved-optical gating (FROG) measurement. a,** Original FROG measurement after de-noising. **b**, FROG mapping retrieval via commercial software. **c,** Pulse shape (black line) and temporal phase (blue line) retrieved from the FROG measurement. The FWHM pulse duration is measured to be 167 fs. The residual chirp *q* is determined to be -0.1672.

**Supplementary Section IV. Ultrafast temporal magnifier**

The UTM is based on the mathematical analogy between the diffraction of an electromagnetic beam and dispersive propagation of an electromagnetic pulse (Figure S9). The total group delay dispersion (GDD), defined as the product of the GVD and the length of the dispersive medium, is analogous to the propagation distance which determines how much a beam is diffracted. An optical lens imparts a spatial quadratic spatial phase that is proportional to *k/f*, where the focal length *f* is a measure of the diffraction required for removal of the phase imparted by the lens. Similarly, one can create a time lens by imparting a quadratic temporal phase (linear frequency chirp *dω/dτ*) to a waveform. A temporal focal GDD, *D*_f_, is defined as the GDD required for removal of the quadratic phase imparted by the time lens. An UTM system is created by cascading an input GDD (*D_1_*), a time lens, and an output GDD (*D_2_*) in proper measure to satisfy the temporal imaging condition:

$\frac{1}{D_{1}}+\frac{1}{D_{2}}=\frac{1}{D_{f}}$ (S2)

A waveform of arbitrary shape passing through this system emerges temporally rescaled with magnification:
$M_{UTM}=-\frac{D_{2}}{D_{1}}$ (S3)

**Figure S9 | The setup for measurement of the performances of the UTM.** WDM: wavelength-division multiplexing; EDFA: erbium-doped fiber amplifier; PD: photodetector; *D_1_*, *D_2_*, and *D*_f_ are the dispersions for the UTM. To increase the SNR and reduce the aberration, the measurement frame rate is sacrificed to 25 MHz by picking 1 pulse out of 10 with an EOM driven by a synchronized AWG (red dashed box). Inset: the spectrum for the input comb, pump and output signal. Inset: The spectra of the UTM. The blue, red, and green is the input comb signal, pump and output signal, respectively.

The parameters of the UTM components are shown in the Table S1. To characterize the performance of the UTM system, a transform-limited femtosecond pulse with a bandwidth of 8 nm and a center wavelength of 1543 nm was used as the test input, as shown in Figure S9. The pulsewidth of the test input was independently measured to be 450 fs by a second-harmonic generation autocorrelation setup.

**Table S1 | Parameters of the UTM components.**

**Figure S10 | Characterization of the UTM using a 450 fs input pulse. a**, The magnified output pulse shape, measuring a FWHM width of 54 ps. After demagnification and deconvolution, it corresponds to a temporal resolution of 600 fs. **b**, The optical spectra of the signal (blue), pump (red) and idler (green) after the HNLF. **c**, The output timing (black) and responsivity (blue) as a function of delay between the pump and the signal, measuring a magnification factor of 72 and a FWHM temporal field-of-view of 30 ps for the first UTM. **d**, The output timing (black) and responsivity (blue) as a function of delay between the pump and the signal, measuring a magnification factor of 61 and a FWHM temporal field-of-view of 190 ps for the second UTM with increased SNR and reduced aberration.

The UTM output waveform and optical spectrum are shown in Figures S10a and S10b. We determined the magnification factor and the FWHM temporal field-of-view by measuring the output timing and responsivity as functions of the delay between the signal and the pump (Figures S10c and S10d). The measured magnification factor and FWHM temporal field-of-view are 72 and 30 ps. The magnified output pulsewidth is 54 ps, corresponding to a temporal resolution of 600 fs after demagnification and deconvolution using the formula:

$\tau=\sqrt{\left(\frac{\tau_{T}}{M_{UTM}}\right)^{2}-\tau_{i}^{2}}$ (S4)

where *τ_T_* and *τ_I_* are the widths of the output and input pulses respectively.

**Supplementary Section V. Stability zone comparison between DM-DS and DS generated in conventional uniform microcavity**

**Figure S11 | Total power transmission with each dataset repeated over 20 times.** **a** and **b**, Multi-scan results for uniform microring. **c** and **d**, Multi-scan results for tapered microring. Each of the laser scan measurements are repeated over 20 times. Red lines are average value for every step. The transmissions are normalized by the average values of the S1 step (minimum) and S4 step (maximum).

For every step of the transmission we calculated the average value and normalized the transmission using the average value of the S1 step (minimum) and S4 step (maximum). We can see that the pump detuning of stable single pulse increased by at least 10 times. The average single pulse energy increased by 1/3. Based on the experimental results, we can conservatively note that the dispersion-managed tapered microcavity increases the pump detuning range for stable single pulse and also increases the average pulse energy.

**Table S2 | Multi-scan data of uniform and dispersion-managed microcavities.**

|  | Average transmission | | | | Relative single pulse energy | Double pulse pump detuning (S2) | Single pulse pump detuning (S3) |
| --- | --- | --- | --- | --- | --- | --- | --- |
|  | S1 | S2 | S3 | S4 |  |  |  |
| Uniform ring 1(a) | 0 | 0.433 | 0.707 | 1 | 29.3% | 176 kHz | 28 kHz |
| Uniform ring 2(b) | 0 | 0.506 | 0.7 | 1 | 30% | 128 kHz | 27 kHz |
| Tapered ring 1(c) | 0 | --- | 0.532 | 1 | 46.8% | --- | 430 kHz |
| Tapered ring 2(d) | 0 | --- | 0.5 | 1 | 50% | --- | 467 kHz |

In addition, we measured a uniform microcavity with GVD at -7.45 fs^2^ mm^-1^, very close to the path-averaged GVD of -6.4 fs^2^ mm^-1^ for the dispersion-managed tapered microcavity. Characterized carefully with swept-wavelength interferometry, the GVD is found to be -7.45 fs^2^ mm^-1^ as shown in Figure S12, within 16% of the dispersion-managed microcavity.

**Figure S12 | Measurements and simulations for a uniform microcavity** **with path-averaged GVD of -7.45 fs^2^ mm^-1^. a,** Dispersion measurement of an 88 GHz uniform microcavity. The fitted GVD is -7.45 fs^2^ mm^-1^. **b,** Total power transmission as the pump frequency is scanned across a cavity resonance at a speed of 2.1 THz/s for a pump power on-chip at 30 dBm. **c,** Nonlinear numerical simulations of the corresponding soliton stability zone.

We then measure the total power transmission as the pump frequency is scanned across a cavity resonance at a speed of 2.1 THz s^-1^ for a pump power on-chip at 30 dBm, shown in Figure S12b. Compared with the main manuscript Figure 3a, the comb stability zone is even smaller than the previous uniform microcavity (at -33.1 fs^2^ mm^-1^) – the characteristic step shows that the *total* stability zone is ≈ 50 kHz, much smaller (≈ 4×) than even the prior uniform microcavity comb (total stability zone of ≈ 200 kHz; and single-soliton stability zone of ≈ 30 kHz). As shown in the main manuscript Figure 3b for our dispersion-managed microcavity comb for the same 30 dBm pump power, the total stability zone is ≈ 340 kHz – our non-uniform microcavity has about 6.8× larger stability zone than the uniform microcavity frequency comb of the same path-averaged GVD.

Furthermore, we note that for the multimode uniform microcavity, when the GVD is close to zero, the higher order dispersion starts to dominate, which will perturb the dynamics of dissipative Kerr soliton. Our designed dispersion-managed microcavity avoids such a problem. We also note prior literature in dispersion-managed fiber cavities for improved performance [S5, S6].

We further substantiate our new measurements with the simulation of the stability zone of a uniform microcavity with GVD of -7.45 fs^2^ mm^-1^, as shown in Figure S12c. While the smaller GVD uniform case (-7.45 fs^2^ mm^-1^) can support the single-soliton to higher pump powers (of 120 mW) than the higher GVD uniform case (of -33.1 fs^2^/mm), its stable pump power is still 2× worse than our dispersion-managed microcavity comb (and 6.8× larger stability zone in terms of detuning). This proves that our dispersion-managed microcavity is more resistant to breather soliton instability -- at larger detunings and at high pump powers – hence it effectively increases the operating range and pulse energy of the frequency microcombs.

**Supplementary Section VI. Effect of periodically-varying nonlinearity**

With the periodically-varying effective cross-sectional mode area, the nonlinear also varies along the ring waveguide. Thus we have included this periodically-varying nonlinearity in our nonlinear pulse simulations. Through the periodically-varying ring waveguide geometry, the dispersion and nonlinearity are intrinsically coupled together via the waveguide cross-sectional area, contributing to the nonlinear dynamics.

In order to isolate only the dispersion management effect, we re-run another set of simulations where the dispersion varies along the cavity while the nonlinearity is constant along the ring waveguide propagation. Figure S13 below shows the new results, on output pulsewidths. This shows that the nonlinearity indeed has small influence, and the dispersion variation dominates the dynamics.

**Figure S13 | Comparison simulations of the temporal intensity and phase** for: **a,** varied nonlinear coefficient along the cavity, and **b,** uniform nonlinear coefficient. In both cases, the GVD is spatially varying periodically, matching our cavity design. The dominating pulse dynamics arises from the periodically-varying GVD.

Supplementary information accompanies the manuscript on the Light: Science & Applications website (http://www.nature.com/lsa/)

**Supplementary References:**

[S1] C. Bao and C. Yang, Stretched cavity soliton in dispersion-managed Kerr resonators, *Phys. Rev. A* **92**, 023802 (2015).

[S2] J. K. Kang, M. Erkintalo, S. G. Murdoch, and S. Coen, Observation of dispersive wave emission by temporal cavity solitons, *Optics Lett.* **39**, 5503 (2014).

[S3] M. Conforti, A. Mussot, A. Kudlinski, and S. Trillo, Modulational instability in dispersion oscillating fiber ring cavities, *Optics Lett.* **39**, 4200 (2014).

[S4] S. Coen, H. G. Randle, T. Sylvestre, and M. Erkintalo, Modeling of octave-spanning Kerr frequency combs using a generalized mean-field Lugiato–Lefever model, *Optics Lett.* **38**, 37 (2013).

[S5] See for example, *G. P. Agrawal, Nonlinear Fiber Optics Fifth Edition Nonlinear Fiber Optics (2007)* page 156.

[S6] Y. Chen and H. A. Haus, Dispersion-managed solitons with net positive dispersion, *Opt. Lett.* **23**, 1013 (1998).
